# Computed Tomography Angiography and Conventional Angiography for the Diagnosis and Treatment of Non-variceal Gastrointestinal Bleeding at a Tertiary Cancer Center

**DOI:** 10.7759/cureus.51031

**Published:** 2023-12-24

**Authors:** Jonathan Kessler, Richard Pham, Frederico Pedersoli, Huiyan Ma, F Edward Boas, Trilokesh D Kidambi

**Affiliations:** 1 Department of Radiology, City of Hope Comprehensive Cancer Center, Duarte, USA; 2 School of Medicine, University of California Riverside School of Medicine, Riverside, USA; 3 Department of Epidemiology and Statistics, City of Hope Comprehensive Cancer Center, Duarte, USA; 4 Department of Medicine, Division of Gastroenterology, City of Hope Comprehensive Cancer Center, Duarte, USA

**Keywords:** ct angiography, oncology, gastrointestinal bleeding, embolization, angiography

## Abstract

Introduction: To evaluate the diagnostic value of computed tomography angiography (CTA) and conventional angiography (CA) and the therapeutic value of transarterial embolization for acute gastrointestinal bleeding in patients with malignancy.

Methods: A retrospective review of 100 patients who underwent CTA and/or CA for gastrointestinal bleeding at a comprehensive cancer center between the years 2011-2021 was performed. Clinical and patient outcome data were collected and analyzed using Kruskal-Wallis tests for continuous variables and chi-square tests or Fisher’s exact tests (whichever is appropriate) for categorical variables in univariate analysis. All tests were two-sided at a significance level of 0.05. Analyses were performed using SAS version 9.4 (SAS Institute, Cary, NC).

Results: Fifty-two percent of our patients underwent CTA alone, 29% underwent CA alone, and 19% underwent both procedures. Overall, CTA was positive in 11.3% (8/71) of patients and CA was positive in 22.9% (11/38) of patients. Of patients who underwent both studies, 52.6% (10/19) were positive for both. ICU admission was associated with CTA and/or CA positivity (p=0.015). Of 48 patients with data for embolization, 50% of patients underwent transarterial embolization for bleeding, 11 patients had identifiable bleeding on CA, and 13 patients underwent prophylactic embolization at the site of suspected bleeding. Rebleeding following embolization was found in 33.3% (8/24) of patients, including six patients who underwent prophylactic embolization and two patients who were treated for visualized bleeding.

Conclusion: CTA and CA are two critical studies for patients with GI bleeding and a history of malignancy. Neither alone can effectively exclude an identifiable source of bleeding. In patients with a history of malignancy, transarterial embolization may be an effective treatment of both angiographically visible and occult sources of GI bleeding.

## Introduction

Nonvariceal acute gastrointestinal (GI) bleeding (GIB) is a common problem among cancer patients that results in significant morbidity and mortality [1,2]. This may result from direct tumor-related bleeding complications such as primary GI neoplasms or direct invasion of intra-abdominal tumors or metastases, or treatment-related complications such as bleeding diatheses, enteritis, and graft-versus-host disease. Diagnosis and treatment are particularly challenging in this population given their often frail status and complex medical conditions. Furthermore, unlike other scenarios with GIB, endoscopic therapy may only be effective in 12-27% of patients [3]. Computed tomography angiography (CTA) has emerged as the first-line modality for diagnosis of endoscopically occult GIB with a reported sensitivity of 90% and specificity of 92% in the general population [4]. However, in cases where no source of bleeding is identified or in cases where bleeding is not controlled with non-invasive measures, catheter-based angiography (CA) is often indicated. CA can detect bleeding at a rate of 0.5 mL/min and allows for direct embolization of bleeding vessels in appropriate patients [5]. However, data on the accuracy of both of these studies in an oncologic population are limited. Furthermore, while selective embolization is routinely employed in the general population with endoscopically untreatable GIB, patients with underlying malignancy may respond poorly to treatment [6,7]. Thus, the aims of this study were to evaluate the utility of CTA and CA in a population of patients with malignancy presenting with GIB and to assess the utility of transarterial embolization.

## Materials and methods

Study design

A retrospective review was performed of all patients who underwent either CTA or CA for GIB at a comprehensive cancer center between the years 2011 and 2021. Institutional review board approval was obtained. Patients were excluded if variceal or non-bowel sources of bleeding were identified. Demographic data, and clinical data such as pre- and post-treatment laboratory data, imaging, endoscopic, embolization findings, discharge, and mortality data were collected by manual chart review. All subjects had clinically evident GIB. The sensitivity calculation of both CA and CTA was assessed in reference to the entire study group. Rebleeding was defined as any further decrease in hematocrit in the setting of further clinical evidence of GIB, such as hematemesis, melena, or hematochezia. Hemodynamic instability was defined as sustained tachycardia or systolic hypotension clinically determined to be related to blood loss anemia.

CTA technique

All images were acquired on a 64-detector row CT system (GE Healthcare, Chicago, IL). No oral contrast was administered. Initial non-contrast images were obtained through the abdomen and pelvis. Moreover, 125 mL of intravenous contrast (Bracco, Milan, Italy) was then administered, and using automatic bolus triggering situated over the abdominal aorta, images were then obtained through the abdomen and pelvis in the arterial, venous (90-100 seconds post-injection), and delayed phases (180 seconds post-injection). Technical parameters included tube rotation of 0.5°, a thickness of 2.5 mm x 2 mm, kV 120, tube modulation max of ma 800, and 0.625 mm acquisition with axial, sagittal, coronal, and MIP reconstructions. All images were initially interpreted by a board-certified radiologist. All images were then reviewed for the presence or absence of bleeding by a board-certified diagnostic/interventional radiologist (JK).

CA and embolization technique

All CA was performed by fellowship-trained interventional radiologists. Standard femoral access was performed, and celiac, SMA, and IMA angiography was performed based on the suspected site of bleeding from the clinical presentation, endoscopic and CTA findings with standard 5F base catheters, and digital subtraction angiography. Further selective angiography was performed with 2-2.8 F microcatheters based on the initial angiographic findings and the stated clinical factors. CA was considered positive if images demonstrated active contrast extravasation, vessel pseudoaneurysm, or other vascular abnormalities such as focal contrast blush. When indicated, selective embolization was performed based on physician discretion.

Statistical analysis

Patients’ characteristics were shown in the contingency table and were compared by factors of interest using Kruskal-Wallis tests for continuous variables and chi-square tests or Fisher’s exact tests (whichever is appropriate) for categorical variables in univariate analysis. All tests were two-sided at a significance level of 0.05. Overall survival (OS) for patients with and without embolization performed was examined using Kaplan-Meier curves and log-rank test.

We did not adjust P values for multiple comparisons as these analyses were considered exploratory. Analyses were performed using SAS version 9.4 (SAS Institute, Cary, NC).

## Results

Findings on CTA and CA are shown in Table 1.

**Table 1:** Computed tomography angiography (CTA) and conventional angiography (CA) in 100 cancer patients

	No. of patients with test(s)	No. patients with positive results	% positive
Overall			
CTA and/or CA	100	17	17%
CTA	71	8	11.3%
CA	48	11	22.9%
By test type			
CTA alone	52	1	1.9%
CA alone	29	6	20.7%
Both CTA and CA (N=19)	19	10	52.6%
Positive for both CTA and CA		2	
Only CTA positive		5	
Only CA positive		3	

Of patients who had CTA and/or CA, 17% (17/100) had a positive study, and 52.6% (10/19) of the patients who underwent both CTA and CA had a positive result. Patient demographics and clinical data, organized by study result, are shown in Table 2, as well as clinical factors associated with a positive study.

**Table 2:** Patient’s characteristics overall and positivity of computed tomography angiography (CTA) or conventional angiography (CA) in 100 cancer patients *P value estimated from the Kruskal-Wallis test, except where otherwise noted. †P value estimated from the chi-square test or Fisher’s exact test, whichever is appropriate. Abbreviations: HCT, hematocrit test; WBC, white blood count; ANC, absolute neutrophil count; INR, international normalized ratio; EGD, esophagogastroduodenoscopy; ICU, intensive care unit; GVHD, graft vs. host disease

	Overall	By CTA/CA result		
		Negative	CTA or CA positive	P value^*^
Patient age, years				0.690
Median (range)	63 (12-90)	63 (12-90)	66 (21-83)	
N	100	83	17	
Primary diagnosis				0.514^†^
Solid tumors	60 (60%)	51 (61.4%)	9 (52.9%)	
Hematological malignancies	40 (40%)	32 (38.6%)	8 (47.1%)	
HCT trough (%)				0.203
Median (range)	25 (10-47)	25 (13-47)	24 (10-34)	
N	96	80	16	
Creatine peak (mg/dL)				0.974
Median (range)	1 (0-5)	1 (0-5)	1 (0-2)	
N	98	81	17	
WBC count (K/μL)				0.852^†^
<4.5	48 (50.0%)	39 (49.4%)	9 (52.9%)	
4.5-11	36 (37.5%)	31 (39.2%)	5 (29.4%)	
>11	12 (12.5%)	9 (11.4%)	3 (17.6%)	
Unknown	4	4		
ANC trough (K/μL)				0.999^†^
<=1500	22 (26.2%)	18 (26.1%)	4 (26.7%)	
>1500	62 (73.8%)	51 (73.9%)	11 (73.3%)	
Unknown	16	14	2	
INR peak				0.359
Median (range)	1 (1-4)	1 (1-4)	1 (1-2)	
History of stem cell transplant				0.261
No	22 (56.4%)	19 (61.3%)	3 (37.5%)	
Yes	17 (43.6%)	12 (38.7%)	5 (62.5%)	
Unknown	1	1		
Graft-versus-host-disease				0.999
No	2 (11.8%)	2 (16.7%)	0 (0%)	
Yes	15 (88.2%)	10 (83.3%)	5 (100%)	
Embolization performed				0.066^†^
No	24 (24%)	19 (59.4%)	5 (31.3%)	
Yes	24 (24%)	13 (40.6%)	11 (68.8%)	
Unknown	52	51	1	
EGD				0.524^†^
No	36 (38.3%)	31 (39.7%)	5 (31.3%)	
Yes	58 (61.7%)	47 (60.3%)	11 (68.8%)	
Unknown	6	5	1	
Lower endoscopy				0.259^†^
No	56 (56.6%)	49 (59%)	7 (43.8%)	
Yes	43 (43.4%)	34 (41%)	9 (56.3%)	
Unknown	1		1	
Endoscopic intervention to address bleeding				1.000^†^
No	35 (50%)	29 (50%)	6 (50%)	
Yes	35 (50%)	29 (50%)	6 (50%)	
Unknown	30	25	5	
Hemodynamically unstable				0.250^†^
No	86 (86%)	73 (88%)	13 (76.5%)	
Yes	14 (14%)	10 (12%)	4 (23.5%)	
ICU admission				0.015^†^
No	53 (53%)	49 (59%)	4 (23.5%)	
Yes	47 (47%)	34 (41%)	13 (76.5%)	
Time from bleeding to hospital discharge, day				0.305
Median (range)	12 (0-410)	10 (0-410)	16 (0-63)	
N	99	82	17	
Time from bleeding to death, months				0.787
Median (range)	2 (0-70)	2 (0-40)	2 (0-70)	
N	72	58	14	
Repeat bleeding after embolization				0.148^†^
No	16 (66.7%)	7 (53.8%)	9 (81.8%)	
Yes	8 (33.3%)	6 (46.2%)	2 (18.2%)	
Unknown	76	70	6	
Prophylactic embolization				<0.0001^†^
No	11 (47.8%)	0 (0%)	11 (100%)	
Yes	12 (52.2%)	12 (100%)	0 (0%)	
Unknown	77	71	6	

There were no associations between age, type of malignancy, laboratory parameters, or results of endoscopic evaluation and CTA or CA results. While ICU admission was associated with positive CTA and/or CA results, hemodynamic instability was not. Subgroup analysis was performed on 40 patients with a history of hematological malignancies, of which 46% underwent hematopoietic stem cell transplant (HSCT) and 89% had evidence of gastrointestinal graft-versus-host-disease (GVHD). There were no statistically significant differences in CTA or CA results according to the presence of HSCT or GVHD.

Characteristics of patients who underwent embolization are shown in Table 3.

**Table 3:** Embolization and characteristics of patients with embolization *Positive for both CTA and CA. Abbreviations: CTA, computed tomography angiography, CA, conventional angiography.

Overall (N=100)	Patients (%)
Embolization	
No	24 (50.0)
Yes	24 (50.0)
Unknown	52
Among patients with embolization (N=24)	
Site of embolization	
Gastroduodenal/gastroepiploic	18
Left gastric	2
Small bowel branch	3
Colon	1
Embolic material	
Gelfoam	8
Microspheres	2
Coils	15
Ethylene vinyl-alcohol copolymer	4
CTA	
Negative	9 (81.8)
Positive	2* (18.2)
Didn’t undergo	13
CA	
Negative	13 (54.2)
Positive	11 (45.8)
Mean repeated number of CA (range)	
Overall	1.1 (0-5)
CA negative	1.1 (0-4)
CA positive	1.2 (0-5)
Prophylactic embolization	
No	11 (45.8)
Yes	13 (54.2)

More than half (54.2%) of the embolizations were performed prophylactically. Upper GIB was addressed with embolization in 20% of the cases, while a colon bleed was addressed in 4% with the specific arteries, as shown in Table 3. Among the 24 patients who underwent embolization, there was no difference in rebleeding rates based on whether CTA or CA studies were positive (p=0.21). Additionally, prophylactic embolization was effective for rebleeding in 53.9% of cases.

Survival analyses were performed stratified by whether patients underwent embolization. While embolization was associated with improved survival, this finding did not reach statistical significance (log rank p-value=0.311).

## Discussion

We report a large experience of non-variceal GIB in patients with a wide range of malignancies requiring CTA and CA at our tertiary referral cancer center, and our primary findings were that neither CTA nor CA was sensitive enough to identify the majority of cases of clinical GIB in this population. The rates of a positive study were substantially reported in a non-oncologic population, highlighting the unique clinical dilemma in managing these patients. Furthermore, embolization either after a positive study or prophylactically appears to be effective in this patient population. However, the rebleeding rate was higher after prophylactic embolization.

Several diagnostic options are available in the setting of GIB and may be impacted by the suspected location of the bleeding site, clinical factors, and available resources. CTA is often the preferred method of evaluation in patients with hemodynamically stable GIB if endoscopy is negative or not feasible [8-10]. It may detect bleeding rates as low as 0.3 mL/min [11]. Accordingly, 81% of the current cohort had a CTA as the primary or exclusive imaging study. CTA was positive in 11.3% of this population. A previous meta-analysis of CTA for GI bleeding has demonstrated a sensitivity of 83% and a specificity of 90% [12]. The substantially lower sensitivity of CTA in the current study may be impacted by several factors. Firstly, many patients in our cohort were known to have clinically intermittent bleeding or were hemodynamically stable, both of which are known to have lower CTA-positive rates than patients with continuous bleeding and hemodynamic instability [13,14]. For example, in a previous study by Chang et al., there was only a 50% sensitivity for CTA in patients with GIB that was unable to be identified on endoscopy and required less than 500 mL blood transfusion to maintain vital signs [15].

Additionally, at our institution, patients with hemodynamically significant bleeding are often taken to emergent angiography, as evidenced by 29% of the cohort having only CA performed. This phenomenon likely results in a negative selection bias against CTA in this population. Nonetheless, 19% of patients had both CTA and CA performed. Among these patients, the sensitivity of CTA was 37% (7/19), and the sensitivity of CA was 26% (5/19). Additionally, of 12 patients with negative CTA, 25% (3/12) were found to have an active site of bleeding on CA. This is particularly important as this may indicate that, in patients with malignancy, CTA may not effectively rule out angiographically treatable bleeding.

Catheter angiography should be performed emergently in hemodynamically unstable patients with endoscopically uncontrollable bleeding [16]. It can detect bleeding at rates as low as 0.5 mL/min and can allow for immediate transcatheter embolization [17]. Much like CTA though, intermittent bleeding, lower number of required transfusions, and hemodynamic stability all correlate with lower rates of positive CA [18,19]. This was confirmed in our cohort, as ICU admission, a likely surrogate for volume and significance of bleeding, was associated with a positive result on either CTA or CA. However, in the current cohort, CA had a sensitivity of only 26%. The reasons for this lower-than-expected sensitivity are uncertain. Given the presence of active malignancy, potential etiologies of bleeding in this population may include a variety of factors that contribute to small vessel or mucosal bleeding, such as direct mucosal tumor involvement, treatment-related enteritis, and coagulopathy. It is possible that, due to the small vessel caliber, these disorders are less likely to demonstrate discrete angiographic findings compared to more common etiologies, such as ulcer or diverticular bleeding commonly seen in a non-oncologic population. However, further study would be required to ascertain the degree to which this contributes to this finding.

Transarterial embolization is a standard treatment for angiographically identified bleeding of both upper and lower GI origins. Clinical success rates from 58% to 91% for UGI and 81-90% for LGI sources [20-24]. However, rebleeding rates have been reported as high as 50% in some populations with the location and etiology of bleeding likely contributing to outcomes [25]. Previous studies have identified rebleeding rates to be higher in oncologic patients as opposed to those with benign sources of bleeding [26]. Additionally, technical failure appears more common in patients with coagulopathy and those with clinical shock and severe anemia [27,28]. This is concordant with the findings in the current study, with a rebleeding rate of 33% in the population. We suspect that the lack of statistically significant associations to anemia or hemodynamic instability may be due to the small sample size.

We also analyzed this cohort for associations between rebleeding rates in patients who had angiographically identifiable bleeding and those treated prophylactically with transarterial embolization for angiographically occult bleeding. Prophylactic embolization has been shown to be clinically effective in 63-85% [29,30]. However, rebleeding rates remain high in this population, and mortality may be as high as 33% [27]. In the current cohort, overall rebleeding followed at 33.3% after embolization, 46% in those with prophylactic embolization, and 18% for patients treated for visualized bleeding. While this finding was not statistically significant, we suspect this was related to the relatively low sample size.

This study is limited by several factors. The retrospective design and small sample sizes restricted our ability to potentially identify other factors that may contribute to differences in this population. Given the retrospective design, clinical evaluation, treatment, and embolization protocols were not standardized. Additionally, given the variety of underlying diseases and malignancies, additional patient-related or disease-specific factors were unable to be assessed for an impact on clinical and imaging outcomes. As such, there may have been selection bias influencing outcomes such as rebleeding rates driven by physician preference. Furthermore, despite a large sample size for this patient population, there was not adequate power to detect statistically significant differences in outcomes. However, we feel these weaknesses reflect the wide spectrum of patient conditions encountered in clinical practice and reflect the complexity of diagnostic and treatment decisions in this patient population. These limitations had the potential to impact the conclusions that can be drawn regarding the sensitivity of imaging modalities and predictive factors. Therefore, additional prospective studies in this complex patient population are warranted.

## Conclusions

Both CTA and CA appear to be valuable diagnostic tests in patients with underlying malignancy and GIB. Neither test in isolation may adequately exclude treatable causes of bleeding. Transarterial embolization remains an effective means of treating patients with malignancy and acute GIB. This may be effective in cases of both angiographically visible bleeding, as well as prophylactically in instances where a discrete site of bleeding is not identified.
